# Interpretable machine learning for predicting early neurological deterioration in symptomatic intracranial atherosclerotic stenosis

**DOI:** 10.3389/fneur.2025.1667119

**Published:** 2025-10-21

**Authors:** Yang Yang, Chunhao Mei, Xiaoning Guo, Jiajia Chen, Tingting Tao, Qingguang Wang

**Affiliations:** ^1^Department of Neurology, Jiangyin Clinical College of Xuzhou Medical University, Jiangyin, Jiangsu, China; ^2^Department of Neurology, Jiangyin Fifth People's Hospital, Jiangyin, Jiangsu, China

**Keywords:** early neurological deterioration, acute ischemic stroke, symptomatic intracranial atherosclerotic stenosis, machine learning, XGBoost mode, SHAP

## Abstract

**Background:**

To develop and validate a machine learning (ML) model for early neurological deterioration (END) risk prediction in patients with symptomatic intracranial atherosclerotic stenosis (SICAS).

**Methods:**

This retrospective cohort study enrolled 557 patients with SICAS between January 2022 and December 2024. Relevant clinical data were collected. Least Absolute Shrinkage and Selection Operator (LASSO) regression selected predictive features from clinical/imaging variables. Five ML algorithms, including Gaussian Naive Bayes (GNB), Gradient Boosting Decision Trees (GBDT), Light Gradient Boosting Machine (LightGBM), Extreme Gradient Boosting (XGBoost), and Logistic Regression (LR), were trained (70% of the data) and validated (30% of the data) using 10-fold cross-validation. Model performance was assessed using the area under the curve (AUC), calibration, and decision curve analysis (DCA). Shapley additive explanations (SHAP) interpreted the feature contributions.

**Results:**

The overall incidence rate of END was 18.13%. The XGBoost model outperformed the other models, achieving a validation AUC of 0.874 (95% CI, 0.809–0.939), a sensitivity of 0.749, a specificity of 0.859, and excellent calibration (deviation: 0.116). DCA indicates the clinical utility of the XGBoost model. Key predictors included the NIHSS score (strongest driver), vascular stenosis severity, Triglyceride Glucose (TyG) index, age, initial systolic blood pressure (SBP), and diabetes. SHAP analysis provided interpretability for the machine learning model and revealed essential factors related to the risk of END in SICAS.

**Conclusion:**

This study demonstrates the potential of ML in predicting END in SICAS patients. The SHAP method enhances the interpretability of the prediction model, providing a practical and implementable solution for the early identification of high-risk patients.

## Introduction

Early neurological deterioration (END), a frequent complication following acute ischemic stroke (AIS) with an estimated incidence ranging from 12.06 to 17.4%, markedly adversely affects patient prognosis (1–3). Research indicates that AIS patients with intracranial atherosclerotic stenosis (ICAS) face a heightened risk of END and are more susceptible to severe disability (4). In China, approximately 46.6% of AIS patients present with ICAS (5), which poses a substantially greater challenge for preventing and managing END.

ICAS contributes to ischemic stroke primarily through several distinct mechanisms, such as *in situ* thrombosis or artery-to-artery embolism, hemodynamic impairment, and branch atheromatous disease (6). These mechanisms are generally not observed in non-ICAS stroke etiologies (7). Furthermore, significant differences have been reported in admission National Institutes of Health Stroke Scale (NIHSS) scores, 90-day functional outcomes, and blood pressure variability (BPV) between patients with symptomatic intracranial atherosclerotic stenosis (SICAS) and those without SICAS (8). Although progress has been made in predicting END in broader stroke populations (9, 10), there remains a lack of dedicated risk prediction tools explicitly tailored to SICAS patients.

Machine learning methods can integrate multi-dimensional clinical data and identify complex non-linear relationships. They have shown significant advantages over traditional models in predicting conditions such as coronary heart disease (CHD) (11), spontaneous intracerebral hemorrhage (12), and ischemic stroke treatment (13). These strengths offer a novel approach to developing more accurate predictive models for END. Leveraging real-world clinical data, this study aimed to construct a machine learning-based predictive model for END risk in SICAS patients and assess the performance of various algorithms.

## Materials and methods

### Study population

This study employed a retrospective, observational cohort design. We enrolled hospitalized patients with AIS who were admitted to the Jiangyin Clinical College of Xuzhou Medical University between January 2022 and December 2024.

Inclusion Criteria were as follows: (1) age ≥ 45 years, (2) Time from symptom onset ≤72 h, (3) The diagnosis meets the diagnostic criteria for acute ischemic stroke (14), and (4) Magnetic Resonance Angiography (MRA) demonstrating stenosis (≥30%) in an intracranial artery segment (C4-M2), with magnetic resonance imaging - diffusion-weighted imaging (MRI-DWI) confirming an acute infarction within the vascular territory supplied by the stenotic artery.

Exclusion Criteria were as follows: (1) age < 45 years, (2) posterior circulation infarction, (3) history of atrial fibrillation (AF) or AF detected on admission electrocardiogram (ECG), (4) NIHSS score > 18 on admission, (5) presence of tandem extracranial stenosis or occlusion in the culprit vessel, and (6) receipt of endovascular therapy.

This study adhered to ethical standards and was approved by the Research Ethics Committee of Jiangyin Clinical College of Xuzhou Medical University (Approval No. 2025-KY019-01).

### Clinical baseline data

The following baseline clinical data were collected from the electronic medical record system:

1. Demographics: age, sex, and body mass index (BMI). BMI was defined as the ratio of a person’s weight (in kilograms) to the square of their height (in meters).
2. Comorbidities: hypertension, diabetes, CHD, hyperlipidemia, and previous stroke.
3. Personal History: smoking history (defined as current smoking or smoking cessation within the past 6 months) and alcohol consumption history (defined as habitual alcohol intake).
4. Clinical assessment: admission NIHSS score, initial systolic blood pressure (SBP), and initial diastolic blood pressure (DBP). The NIHSS scores were assessed by certified neurologists at our center and independently evaluated by a second blinded neurologist. A senior neurologist adjudicated any discrepancies.
5. Laboratory investigations: fasting venous blood samples were collected at 06:00 the following morning and analyzed for white blood cell (WBC) count, platelet (PLT) count, total cholesterol (TC), low-density lipoprotein (LDL), high-density lipoprotein (HDL), triglycerides (TG), and fasting blood glucose (FBG). The triglyceride glucose (TyG) index was calculated using the following formula: TyG index = Ln [TG (mg/dL) × FBG (mg/dL)/2] (15).

### Imaging assessment

Brain MRI and magnetic resonance angiography (MRA) were performed using a 3.0 Tesla Siemens MRI scanner. The acquired sequences included T1-weighted, T2-weighted, fluid-attenuated inversion recovery (FLAIR), and time-of-flight (TOF) MRA images. Intracranial artery stenosis severity was quantified via the Warfarin–Aspirin symptomatic intracranial disease (WASID) criteria (16): stenosis (%) = (narrowest luminal diameter at the lesion site−/−diameter of the proximal normal vessel) × 100. The severity of vascular stenosis is classified as mild (30–50%), moderate (50–70%), and severe or occlusive (> 70% or complete occlusion). If multiple stenoses were present, the data from the most severe stenosis were recorded. MRI-DWI confirmed an acute infarction within the vascular territory supplied by the stenotic artery. Recorded stenosis sites included the internal carotid artery (ICA) segments C4-C7 and the middle cerebral artery (MCA) segments M1-M2. The first radiologist initially evaluated all imaging and then reviewed it by a second, more experienced radiologist; any disagreements were resolved by a senior radiologist at the center.

### Clinical treatment

Treatment modalities were recorded as follows: (1) Receipt of intravenous thrombolysis (IVT). (2) Antiplatelet therapy: Dual antiplatelet therapy (DAPT) or single antiplatelet therapy (SAPT). (3) Receipt of statin therapy.

### Outcome measure

In this study, the primary outcome measure, END, was defined as either a ≥ 2-point increase in the NIHSS total score or a ≥ 1-point increase in the motor items of the NIHSS scale, occurring within 24 h of hospital admission. This threshold was selected because it is a sensitive indicator of poor functional outcomes (17). All NIHSS scores were evaluated by certified and trained neurologists or research nurses at the time of patient admission (baseline) and every 4 h thereafter within 24 h.

### Statistical analysis

Statistical analyses were performed using R (version 3.6.8) and Python (version 3.7). The normality of continuous variables was assessed using the Shapiro–Wilk test. Data are presented as mean ± standard deviation (SD) for normally distributed variables and as median with interquartile range (IQR) for non-normally distributed variables. Categorical variables are presented as counts (percentages) and were compared using the chi-square test. The 95% confidence interval for the model’s performance was estimated from the distribution of scores obtained from the cross-validation folds. Statistical significance was set at *p* < 0.05.

### Machine learning model construction

Variables with >5% missing data were excluded from analysis; variables with ≤5% missingness were imputed using multiple imputation. The dataset was randomly split into a training set and a validation set in a 7:3 ratio. Following the standardization of quantitative features, the Least Absolute Shrinkage and Selection Operator (LASSO) algorithm was applied to the training set to select the most predictive features (features with non-zero coefficients). A 10-fold cross-validation procedure was incorporated during LASSO feature selection to maximize the area under the receiver operating characteristic (ROC) curve (AUC). LASSO is a regularization regression technique commonly used to reduce high-dimensional feature spaces and aid in identifying and selecting optimal clinical predictors for subsequent model building.

The synthetic minority over-sampling technique (SMOTE) was used to address the issue of class imbalance. Five machine learning algorithms were utilized to predict END risk in SICAS patients: Logistic Regression (LR), Light Gradient Boosting Machine (LightGBM), Gradient Boosting Decision Trees (GBDT), Extreme Gradient Boosting (XGBoost), and Gaussian Naive Bayes (GNB). Each model possesses unique advantages: LR is the most traditional and interpretable method in clinical prediction models, and its inclusion helps determine whether more complex machine learning models yield significant performance improvements (18). LightGBM’s computational efficiency makes it an ideal choice for large-scale datasets (19). GBDT serves as the classical implementation of gradient boosting (20). XGBoost, another gradient boosting method, is renowned for its robust and high-performing nature, making it a powerful tool for classification and regression tasks in medical research (21). GNB, based on the Bayesian theorem, offers simplicity and rapid execution, providing a distinct benchmark compared to other complex models based on gradient boosting (22).

For the training set, k-fold cross-validation (*k* = 10) was employed as the resampling technique, and hyperparameter tuning was performed using a grid search. Model discriminatory ability was assessed using ROC curves and precision-recall (PR) curves. The calibration curves were used to calibrate the models. A decision curve analysis (DCA) was performed to estimate the net clinical benefit. Additionally, the performance of each model was evaluated using a confusion matrix, reporting the following metrics: accuracy, sensitivity, specificity, Positive predictive value (PPV), negative predictive value (NPV), F1-score, and Cohen’s kappa coefficient. The F1-score, which is the harmonic mean of precision and recall, is particularly suitable for evaluating model performance on imbalanced datasets. Meanwhile, Cohen’s Kappa coefficient assesses the agreement between model predictions and actual outcomes, accounting for the possibility of random agreement. This makes it a more reliable metric than accuracy alone.

## Results

### Baseline characteristics comparison

**Figure 1:**
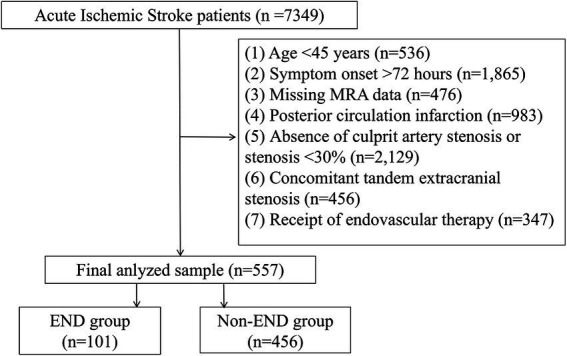
Study flowchart. A total of 557 patients were included in the final analysis.

A total of 557 eligible patients from the Jiangyin Clinical College of Xuzhou Medical University were included in the model (Figure 1). The overall incidence of END was 18.13%. The median age of the participants was 62 years (IQR: 56–70), and 52.96% were male. Compared with the non-END group, the END group was significantly older (*p* = 0.028), had a higher admission NIHSS score (*p* < 0.001), included more patients with a history of diabetes mellitus (*p* < 0.001), and showed elevated SBP (*p* < 0.001). In terms of imaging assessments, the END group demonstrated a significantly greater frequency of severe stenosis or occlusion (*p* < 0.001), as well as a higher prevalence of stenosis in the M1 segment (*p* = 0.030), relative to the non-END group (Table 1).

**Table 1:** Baseline characteristics of patients with and without END (SICAS cohort, Jiangyin Clinical College, 2022–2024).

Characteristic	END			*p*-value
	Overall *N* = 557	Non-END group *N* = 456	END group *N* = 101	
Age, median (Q1, Q3)	62 (56, 70)	61 (56, 69)	65 (56, 71)	0.028^1^
Sex, *n* (%)				0.321^2^
Female	262 (47.0%)	219 (48.0%)	43 (42.6%)	
Male	295 (53.0%)	237 (52.0%)	58 (57.4%)	
Hypertension, *n* (%)				0.315^2^
No	195 (35.0%)	164 (36.0%)	31 (30.7%)	
Yes	362 (65.0%)	292 (64.0%)	70 (69.3%)	
Diabetes, *n* (%)				0.002^2^
No	370 (66.4%)	316 (69.3%)	54 (53.5%)	
Yes	187 (33.6%)	140 (30.7%)	47 (46.5%)	
CHD, *n* (%)				0.635^2^
No	337 (60.5%)	278 (61.0%)	59 (58.4%)	
Yes	220 (39.5%)	178 (39.0%)	42 (41.6%)	
Hyperlipidemia, *n* (%)				0.099^2^
No	284 (51.0%)	240 (52.6%)	44 (43.6%)	
Yes	273 (49.0%)	216 (47.4%)	57 (56.4%)	
Stroke, *n* (%)				0.669^2^
No	455 (81.7%)	374 (82.0%)	81 (80.2%)	
Yes	102 (18.3%)	82 (18.0%)	20 (19.8%)	
Smoking, *n* (%)				0.217^2^
No	387 (69.5%)	322 (70.6%)	65 (64.4%)	
Yes	170 (30.5%)	134 (29.4%)	36 (35.6%)	
Alcohol, *n* (%)				0.684^2^
No	379 (68.0%)	312 (68.4%)	67 (66.3%)	
Yes	178 (32.0%)	144 (31.6%)	34 (33.7%)	
BMI, median (Q1, Q3)	21.85 (19.59, 23.91)	21.78 (19.53, 23.92)	22.09 (20.07, 23.90)	0.487^1^
NIHSS score, median (Q1, Q3)	5 (3, 7)	5 (3, 7)	6 (4, 9)	<0.001^1^
Vascular stenosis severity, *n* (%)				<0.001^2^
Mild	265 (47.6%)	241 (52.9%)	24 (23.8%)	
Moderate	179 (32.1%)	147 (32.2%)	32 (31.7%)	
Severe or occlusion	113 (20.3%)	68 (14.9%)	45 (44.6%)	
Stenosis site, *n* (%)				0.030^2^
C4-C7	231 (41.5%)	193 (42.3%)	38 (37.6%)	
M1	183 (32.9%)	139 (30.5%)	44 (43.6%)	
M2	143 (25.7%)	124 (27.2%)	19 (18.8%)	
SBP (mmHg), *n* (%)				<0.001^2^
<140	159 (28.5%)	131 (28.7%)	28 (27.7%)	
140–160	240 (43.1%)	213 (46.7%)	27 (26.7%)	
>160	158 (28.4%)	112 (24.6%)	46 (45.5%)	
DBP (mmHg), *n* (%)				0.318^3^
<90	235 (42.2%)	198 (43.4%)	37 (36.6%)	
90–110	305 (54.8%)	243 (53.3%)	62 (61.4%)	
>110	17 (3.1%)	15 (3.3%)	2 (2.0%)	
TC (mmol/L), mean ± SD	4.64 ± 0.59	4.63 ± 0.59	4.71 ± 0.58	0.172^4^
HDL (mmol/L), median (Q1, Q3)	1.11 (1.01, 1.24)	1.11 (1.00, 1.24)	1.08 (1.01, 1.21)	0.385^1^
LDL (mmol/L), mean ± SD	3.07 ± 0.56	3.05 ± 0.55	3.15 ± 0.57	0.117^4^
WBC (× 10^9^/L), median (Q1, Q3)	7.76 (5.98, 9.69)	7.86 (6.12, 9.74)	6.99 (5.55, 9.58)	0.184^1^
PLT (× 10^9^/L), mean ± SD	204 ± 51	203 ± 51	210 ± 55	0.218^4^
TyG index, median (Q1, Q3)	9.01 (8.81, 9.17)	9.01 (8.81, 9.16)	9.04 (8.82, 9.22)	0.107^1^
Antiplatelet, *n* (%)				0.701^2^
DAPT	34 (6.1%)	27 (5.9%)	7 (6.9%)	
SAPT	523 (93.9%)	429 (94.1%)	94 (93.1%)	
IVT, *n* (%)				0.488^2^
No	467 (83.8%)	380 (83.3%)	87 (86.1%)	
Yes	90 (16.2%)	76 (16.7%)	14 (13.9%)	
Statins, *n* (%)				0.761^3^
No	19 (3.4%)	15 (3.3%)	4 (4.0%)	
Yes	538 (96.6%)	441 (96.7%)	97 (96.0%)	

^1^Wilcoxon rank sum test; ^2^Pearson's Chi-squared test; ^3^Fisher's exact test; ^4^Welch Two-Sample t-test.

BMI, body mass index; CHD, coronary heart disease; NIHSS, National Institutes of Health Stroke Scale; WBC, white blood cell; PLT, platelet; TC, total cholesterol; LDL, low-density lipoprotein; HDL, high-density lipoprotein; TyG index, triglyceride-glucose index; DAPT, dual antiplatelet therapy; SAPT, single antiplatelet therapy. Mild stenosis, 30–50%; moderate stenosis, 50–70%; severe or occlusion, 70–100%.

### Feature selection for machine learning models

**Figure 2:**
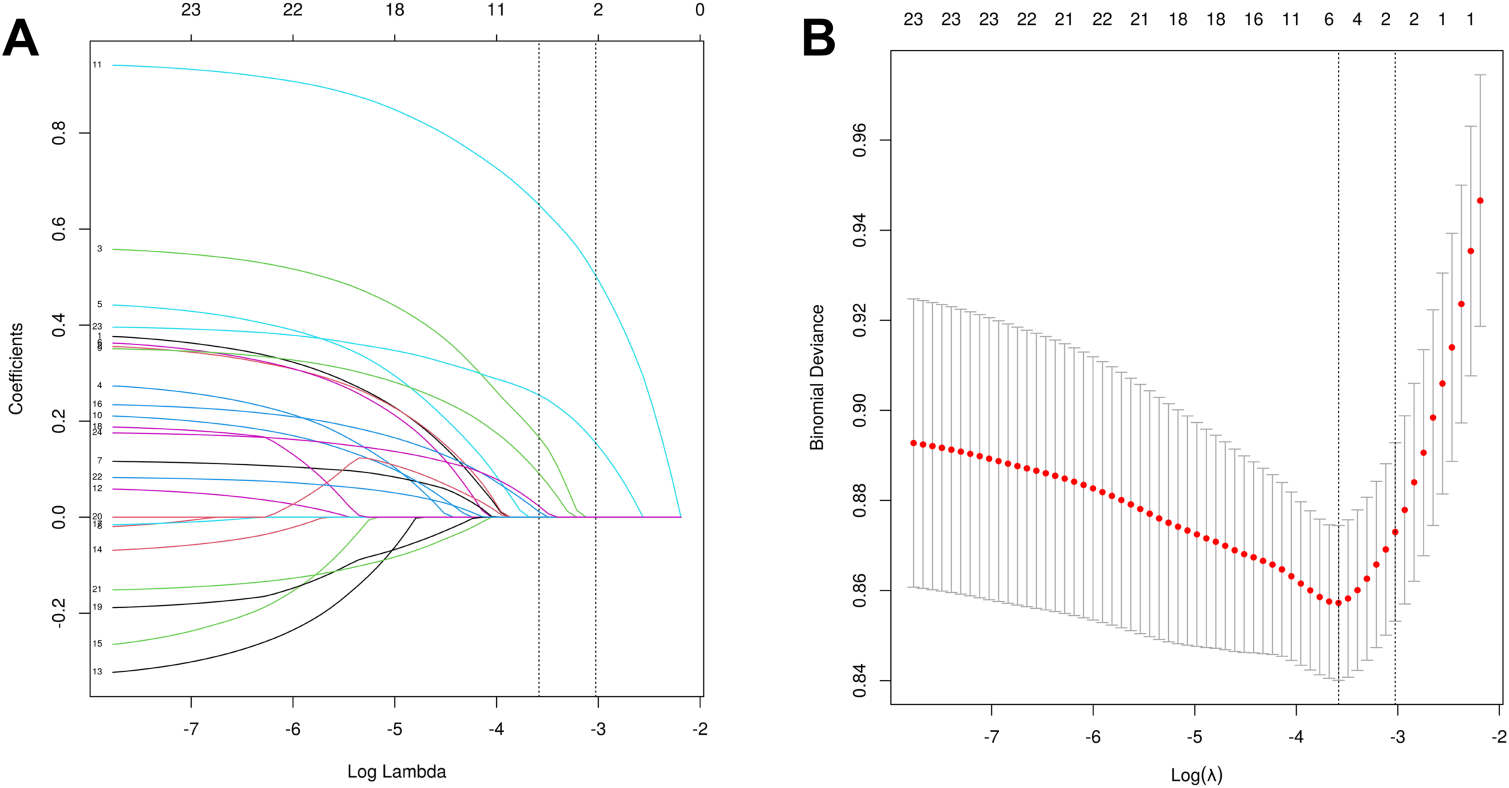
Feature selection using LASSO regression analysis. Panel **(A)** displays the coefficient trajectories of the 24 candidate features across varying penalty parameter (*λ*) values, illustrating the evolution of LASSO coefficients during regularization. Panel **(B)** presents the coefficient profiles of all 24 features across the log(λ) sequence in the LASSO model, with vertical dashed lines indicating the optimal λ values at the minimum mean squared error (λ = 0.028) and one standard error above the minimum (λ = 0.049). The optimal λ value (λ = 0.028) yielded six variables with non-zero coefficients.

LASSO regression analysis was performed on the training dataset encompassing 24 variables. The optimal *λ* value, indicated by the vertical dashed line in Figure 2B, was λ = 0.028. This λ value corresponded to the retention of six predictive features in the model: age, diabetes, initial SBP, admission NIHSS Score, TyG index, and vascular stenosis severity (Figure 2).

### Machine learning models

**Figure 3:**
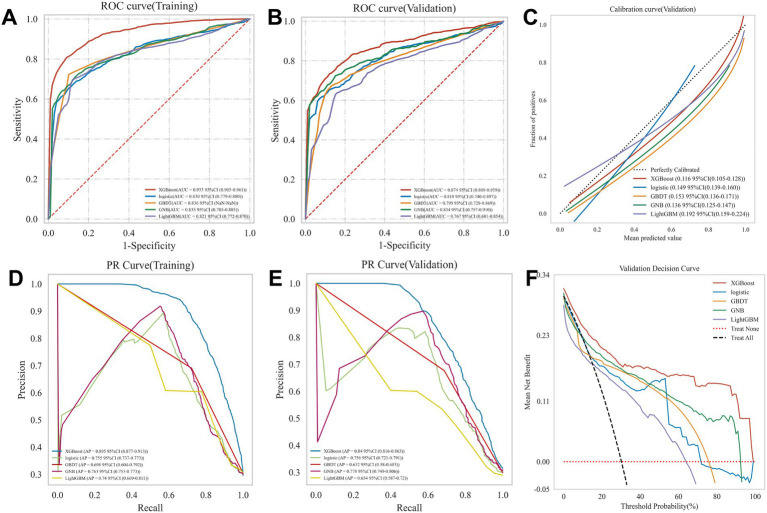
Evaluation of the machine learning model. **(A)** ROC curves in the training set; **(B)** ROC curves in the validation set; **(C)** Calibration curve in the validation set; **(D)** Precision-recall (PR) curve in the training set; **(E)** PR curve in the validation set; **(F)** Decision curve analysis (DCA) in the validation set.

We evaluated the five models using accuracy, sensitivity, specificity, PPV, NPV, F1-score, and Cohen’s kappa coefficient. All five machine learning models achieved mean accuracy exceeding 0.80 in the training set, with their predictive capability further validated in the independent validation set (Table 2). Figures 3A,B demonstrate that the XGBoost algorithm exhibited superior performance and stability in both the training (ROC-AUC 0.933, 95% CI 0.905–0.961) and validation (ROC-AUC 0.874, 95% CI 0.809–0.939) sets. The precision-recall curves (Figures 3D,E) confirmed XGBoost’s optimal performance and robust generalizability, with an area under the precision-recall curve (AUPRC) value of 0.895 (95% CI 0.877–0.913) in the training set and 0.840 (95% CI 0.816–0.863) in the validation set. The calibration plot for the validation set (Figure 3C) indicated a minimal deviation (0.116, 95% CI 0.105–0.128) between predicted probabilities and observed event rates for END risk in the XGBoost model. Decision curve analysis (Figure 3F) revealed that the XGBoost model provided a significantly greater net clinical benefit than the other four models across the threshold probabilities. The ablation analysis (Table 3) showed that the complete model outperformed the model excluding the TyG index across all metrics. Specifically, the AUC decreased from 0.874 to 0.840, and the F1-score dropped from 0.728 to 0.698, confirming that while the TyG index is a valuable predictive feature, it is not the sole determinant of model performance.

**Table 2:** Performance metrics of machine learning models in the validation set.

Model	AUC (95% CI)	Accuracy (95% CI)	Sensitivity (95% CI)	Specificity (95% CI)	PPV (95% CI)	NPV (95% CI)	F1-score (95% CI)	Kappa (95 %CI)
XGBoost	0.874 (0.809–0.939)	0.824 (0.806–0.843)	0.749 (0.721–0.777)	0.859 (0.835–0.883)	0.71 (0.666–0.754)	0.882 (0.873–0.892)	0.728 (0.696–0.760)	0.598 (0.554–0.642)
LR	0.819 (0.740–0.897)	0.834 (0.823–0.844)	0.602 (0.529–0.675)	0.93 (0.895–0.965)	0.818 (0.753–0.884)	0.849 (0.832–0.866)	0.68 (0.649–0.711)	0.572 (0.543–0.601)
GBDT	0.799 (0.729–0.869)	0.825 (0.807–0.843)	0.659 (0.608–0.710)	0.896 (0.875–0.917)	0.734 (0.701–0.767)	0.861 (0.837–0.884)	0.691 (0.664–0.717)	0.57 (0.532–0.608)
GNB	0.834 (0.757–0.910)	0.817 (0.798–0.836)	0.674 (0.616–0.732)	0.88 (0.848–0.912)	0.67 (0.609–0.730)	0.861 (0.840–0.881)	0.69 (0.653–0.727)	0.561 (0.514–0.608)
LightGBM	0.767 (0.681–0.854)	0.795 (0.764–0.826)	0.623 (0.559–0.686)	0.864 (0.819–0.910)	0.818 (0.753–0.884)	0.85 (0.829–0.872)	0.636 (0.595–0.677)	0.495 (0.435–0.554)

XGBoost, extreme gradient boosting; LR, logistic regression; GBDT, gradient boosting decision trees; GNB, Gaussian naive bayes; LightGBM, light gradient boosting machine; AUC, area under the curve; PPV, positive predictive value; NPV, negative predictive value.

**Table 3:** Performance comparison between the complete model and the ablation model (excluding the TyG index).

Model variant	AUC (95% CI)	Accuracy (95% CI)	Sensitivity (95% CI)	Specificity (95% CI)	PPV (95% CI)	NPV (95% CI)	F1-score (95% CI)	Kappa (95% CI)
Complete model	0.874 (0.809–0.939)	0.824 (0.806–0.843)	0.749 (0.721–0.777)	0.859 (0.835–0.883)	0.71 (0.666–0.754)	0.882 (0.873–0.892)	0.728 (0.696–0.760)	0.598 (0.554–0.642)
Ablation model	0.84 (0.77–0.91)	0.789 (0.776–0.801)	0.722 (0.693–0.751)	0.843 (0.818–0.868)	0.685 (0.640–0.730)	0.869 (0.859–0.879)	0.698 (0.665–0.731)	0.561 (0.514–0.608)

### Optimal XGBoost model construction and evaluation

**Figure 4:**
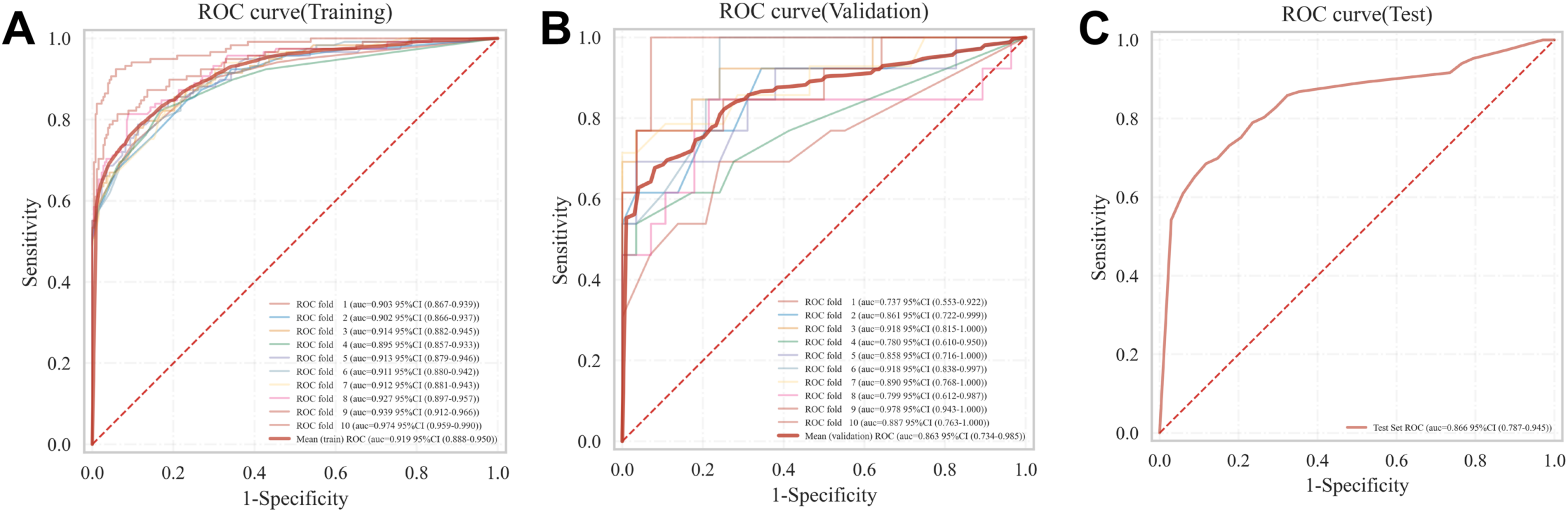
XGBoost model training, validation, and testing. **(A)** Training set ROC and AUC; **(B)** Validation set ROC and AUC. Cross-validation was performed using data from 10% of the patients. Solid lines in different colors represent the 10 distinct results. **(C)** Test set ROC and AUC. Testing results from 30% of the patients.

The XGBoost model was trained using a 10-fold cross-validation. The results demonstrated a mean AUC of 0.919 (95% CI 0.888–0.950) in the training set, a mean AUC of 0.863 (95% CI 0.734–0.985) in the validation set, and a mean AUC of 0.866 (95% CI 0.787–0.945) in the test set (Figures 4A–C), indicating favorable predictive performance of the model.

### Model interpretation

**Figure 5:**
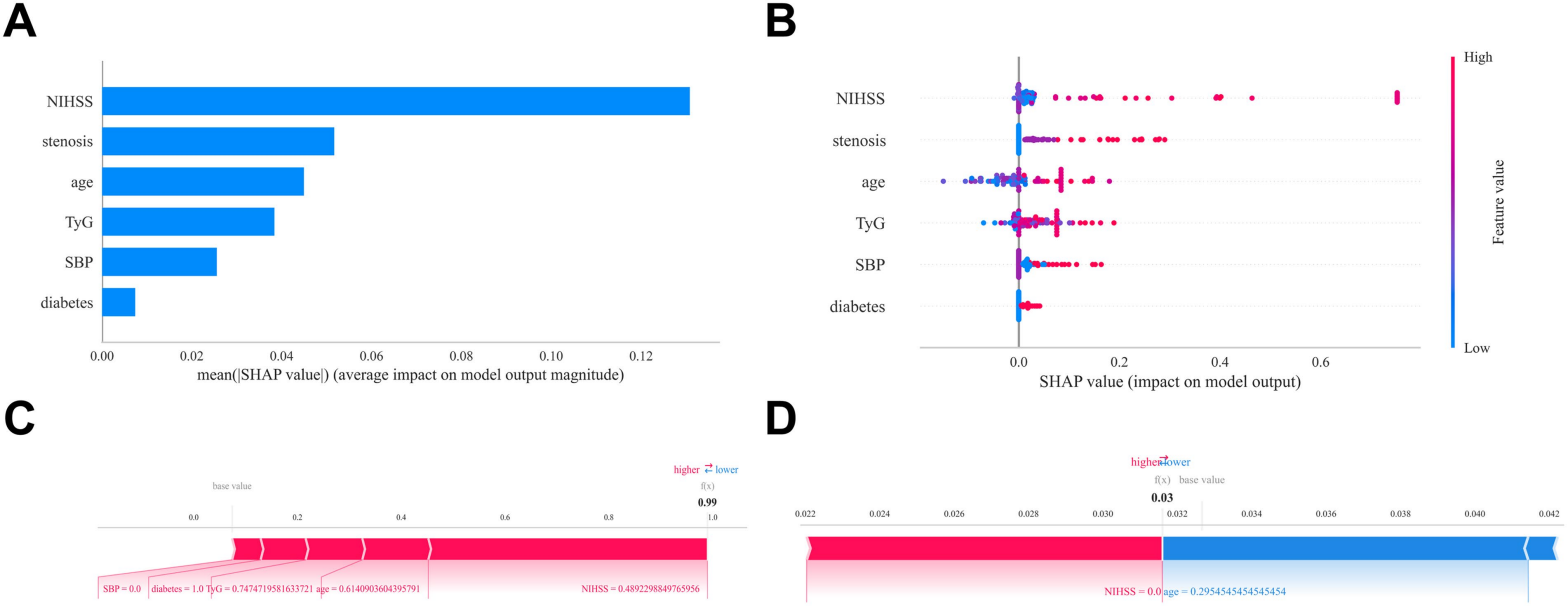
Interpretability analysis of the optimal machine learning model (XGBoost) using SHAP. **(A)** Feature importance matrix plot demonstrating variable contributions to the final predictive model. Stenosis: Vascular stenosis severity. **(B)** SHAP feature attribution plot. Each row represents a feature, with the *x*-axis indicating SHAP values. Red dots denote higher feature values; blue dots indicate lower values. **(C-D)** Individual prediction explanations using SHAP force plots. Red features increase END risk; blue features decrease risk. Arrow length corresponds to effect magnitude—longer arrows represent stronger impacts on prediction outcomes.

SHAP analysis identified the admission NIHSS score as the most influential predictor in the model (Figure 5A). Higher admission NIHSS scores, elevated TyG index, advanced age, severe vascular stenosis, elevated initial SBP, and diabetes were all associated with an increased END risk (Figures 5A,B). Two representative cases further demonstrate the interpretability of the model: Figure 5C illustrates an END patient, while Figure 5D shows a non-END patient.

## Discussion

We unearthed six pivotal predictors of END in patients with SICAS: age, diabetes, TyG index, initial SBP, admission NIHSS score, and the severity of vascular stenosis. To enhance our understanding, we developed a sophisticated machine learning model powered by XGBoost, specifically designed to assess the risk of END in these patients. Remarkably, internal validation underscored the model’s exceptional discriminative ability, strong calibration, and impressive predictive accuracy, positioning it as a vital tool in clinical practice.

### NIHSS score and age

Our model confirms that the admission NIHSS score is the most significant predictor of END in patients with SICAS, which is consistent with existing research (2). The NIHSS is the most widely used neurological assessment tool, effectively evaluating the size of the infarct, neurological status, and functional outcomes in patients with AIS. Since these factors are the strongest predictors of functional outcomes 3 months after a stroke (23), many established models for predicting END consistently include both the admission NIHSS score and the patient’s age (24–26). Therefore, it is essential to perform standardized and thorough NIHSS assessments for all admitted patients, particularly the elderly. This practice will help guide optimized care and early intervention, potentially reducing the risk of END through timely management.

### Vascular stenosis severity

Although both the severity and location of intracranial stenosis showed significant differences between groups, our analysis found that stenosis severity—rather than its location—was a strong predictor of END, second only to NIHSS score. Previous studies have established a link between arterial stenosis or occlusion and END (27). For instance, one prospective multicenter cohort study (28) indicated that ICAS, as opposed to extracranial arterial stenosis (ECAS), is a clear risk factor for END. Additionally, ICAS has been identified as an independent risk factor for END and long-term disability in patients with single subcortical infarcts (29), likely because severe stenosis significantly reduces blood flow beyond the narrowed segment (30). A *post hoc* analysis of the ARAMIS trial (31) suggested that DAPT was associated with a lower risk of END compared to intravenous thrombolysis in minor stroke patients with no or mild stenosis. Conversely, the early use of tirofiban effectively reduced the risk of END in SICAS patients with severe stenosis or occlusion (32). These findings underscore the importance of stenosis severity in predicting the risk of END and support the need for tailored treatments based on the characteristics of stenosis.

### Diabetes and TyG index

Insulin resistance (IR) is a critical mechanism in diabetes that contributes to the formation and rupture of atherosclerotic plaques through various pathways (33), which is also strongly associated with END (34). The TyG index serves as a simple and reliable marker of insulin resistance (35). A high TyG index level is linked to END in patients with AIS (36) and is an independent risk factor for END following thrombolysis (37). There are significant differences in the vascular wall properties between ICAS and ECAS—including aspects such as structure, metabolism, and antioxidant activity—which make ICAS more likely to result in atherosclerosis and plaque instability due to endothelial dysfunction (38, 39). Consequently, the TyG index may be more relevant for predicting ICAS than ECAS (40). Our previous study (41) also confirmed that the TyG index is significantly associated with severe intracranial stenosis and SICAS in nondiabetic patients. These findings highlight the importance of the TyG index and diabetes as significant predictors of END. Our study, along with ablation analysis, confirms that while the TyG index is a valuable predictive feature, it is not the sole determinant of model performance. Meanwhile, the TyG index has limitations when it comes to predicting END within the critical 24-h window. First, its calculation requires fasting conditions. Second, the TyG index reflects a chronic, underlying metabolic state rather than acute events (such as thrombus propagation or hemodynamic fluctuations). Future studies should consider integrating the TyG index with acute-phase biomarkers (for example, inflammatory markers) or imaging features. This combined approach could provide a more comprehensive model for predicting END across different timeframes, thereby enhancing its clinical utility for personalized risk management.

### Initial SBP

Most patients in our study had an initial SBP above 140 mmHg, likely due to reflex hypertension after acute stroke (42). While the ENCHANTED trial (43) showed that intensive BP lowering is safe in general stroke patients, those with ICAS may be at higher risk due to reduced hemodynamic reserve. Optimal BP targets may differ for this group. Current evidence offers mixed insights: a secondary analysis of ENCHANTED (44) found that although intracranial stenosis did not generally affect outcomes from intensive BP control (SBP 120–140 mmHg), patients with severe stenosis had higher END risk. The BP-TARGET trial (45) showed that lowering SBP below 120 mmHg after EVT could increase END risk due to hypoperfusion. Conversely, pre-thrombolysis BP above guidelines (180–185/110 mmHg) is associated with END (46), and high admission SBP (158 vs. 131 mmHg) correlates with END in large artery occlusion (47). In our cohort, the non-END group most often had median SBP levels (140–160 mmHg), while the END group peaked at SBP > 160 mmHg. This unexpected pattern raises the hypothesis that moderate SBP elevation (140–160 mmHg range) might confer protective effects against END risk, a proposition requiring validation through prospective multicenter clinical investigations.

### XGBoost model and SHAP framework

In this study, the XGBoost model demonstrates significant advantages over traditional LR, extending beyond just a modest improvement in discriminatory performance. Importantly, XGBoost excels at capturing complex nonlinear relationships and interaction effects among predictor variables related to END risk. Unlike LR, ensemble algorithms like XGBoost automatically identify and model these intricate patterns, potentially providing a more accurate understanding of the pathophysiological mechanisms associated with END. Integrating the XGBoost model with the SHAP framework is particularly important, as it greatly enhances the model’s clinical interpretability. This integration allows for a transition from population-level predictions to individualized assessments. The ability to clarify specific risk-driving factors offers clear and transparent insights for clinical decision-making, which aids in developing more targeted monitoring and intervention strategies—something traditional LR models often struggle to achieve. Therefore, the combination of XGBoost and SHAP not only improves predictive accuracy but also serves as a practical and actionable tool for personalized risk management of high-risk patients.

### Limitations

First, the retrospective design, reliance on a single-center data source, and restrictions based on age, NIHSS scores, and anterior circulation infarction may limit the generalizability of our findings to broader populations, highlighting the need for validation through larger, prospective, multicenter studies. Second, this study did not explicitly differentiate between hemorrhagic and ischemic END; therefore, the impact of symptomatic intracranial hemorrhage (sICH) on those events was not adequately evaluated. Third, using MRA as a non-invasive vascular imaging tool has limitations; it is not very effective at distinguishing the causes of stenosis (such as differentiating atherosclerosis from arterial dissection) and has relatively lower sensitivity for detecting mild stenosis. This disadvantage may impact the accuracy of the model’s input features and, in turn, somewhat undermine the reliability of our conclusions. Fourth, we conducted only internal validation, and external validation is needed further to strengthen the robustness of our machine-learning predictive model. Additionally, our analysis may not have included certain critical variables, such as broader sociodemographic factors and detailed in-hospital therapeutic regimens, which could have significantly influenced patient outcomes.

## Conclusion

This study demonstrates the potential of ML in predicting END in SICAS patients. The SHAP method enhances the interpretability of the prediction model, providing a practical and implementable solution for the early identification of high-risk patients.

## Data Availability

The raw data supporting the conclusions of this article will be made available by the authors, without undue reservation.
